# Supplementary material for: Application of Pressurized Liquid Extractions to Obtain Bioactive Compounds from Tuber aestivum and Terfezia claveryi
Source: Foods. 2022 Jan 23;11(3):298. doi: 10.3390/foods11030298 (PMC8834127; doi:10.3390/foods11030298)
Supplement: Supplementary file 1 [file foods-11-00298-s001.zip › foods-1552587-supplementary.pdf]

foods

Supplementary material

Table S1. Response surface methodology equations

Equation	*Terfezia claveryi*	*Tuber aestivum*
Yield (water)	*39.3033 - 0.119902 × T^a + 0.694491 × Time + 0.00150047 × T^a^2 - 0.0004 × T^a × Time - 0.0157474 × Time^2*	*8.25354 - 0.0381602 × T^a - 0.187612 × Time + 0.000670819 × T^a^2 + 0.00249231 × T^a × Time + 0.00181895 × Time^2*
Yield (ethanol)	*8.25354 - 0.0381602 × T^a - 0.187612 × Time + 0.000670819 × T^a^2 + 0.00249231 × T^a × Time + 0.00181895 × Time^2*	*-4.89654 + 0.142758 × T^a + 0.143395 × Time - 0.0000415447 × T^a^2 + 0.000618462 × T^a × Time - 0.00480337 × Time^2*
B-glucan content	*13.3066 + 0.327238 × T^a - 0.137132 × Time - 0.00122087 × T^a^2 + 0.00227385 × T^a × Time - 0.00197221 × Time^2*	*13.1984 - 0.283868 × T^a + 0.0762111 × Time + 0.00153217 × T^a^2 + 0.00265538 × T^a × Time - 0.00666611 × Time^2*
Chitin content	*2.27214 + 0.0569907 × T^a + 0.155956 × Time - 0.0000943631 × T^a^2 - 0.000221538 × T^a × Time - 0.00245558 × Time^2*	*4.90825 - 0.0252145 × T^a + 0.166011 × Time + 0.000311367 × T^a^2 + 0.000104615 × T^a × Time - 0.00361263 × Time^2*
Protein conent	*2.97738 - 0.00087711 × T^a - 0.0813725 × Time - 0.0000253504 × T^a^2 + 0.000264615 × T^a × Time + 0.00139453 × Time^2*	*0.986053 - 0.000356317 × T^a + 0.00166165 × Time + 0.00000498287 × T^a^2 - 0.0000246154 × T^a × Time + 0.000166737 × Time^2*
TPC content	*5.87348 - 0.0372458 × T^a + 0.0283163 × Time + 0.000127624 × T^a^2 - 0.0000369231 × T^a × Time + 0.000218947 × Time^2*	*6.14634 - 0.0553149 × T^a + 0.0212034 × Time + 0.000222049 × T^a^2 + 0.0000153846 × T^a × Time - 0.000747789 × Time^2*
Total sterols content	*4.33445 - 0.0762079 × T^a + 0.0213272 × Time + 0.00062261 × T^a^2 + 0.000409231 × T^a × Time - 0.000540632 × Time^2*	*-14.4301 + 0.367708 × T^a - 0.121301 × Time - 0.00138399 × T^a^2 - 0.000129231 × T^a × Time + 0.00424084 × Time^2*
